# Evaluation of the Antioxidant and Antimicrobial Activities of Ethyl Acetate Extract of *Saccharomyces cerevisiae*

**DOI:** 10.17113/ftb.59.02.21.6658

**Published:** 2021-06

**Authors:** Essam A. Makky, Manaf AlMatar, Mahmood H. Mahmood, Ooi Wei Ting, Wong Zi Qi

**Affiliations:** Faculty of Industrial Sciences and Technology, Universiti Malaysia Pahang (UMP), Gambang, 26300 Kuantan, Malaysia

**Keywords:** *Saccharomyces cerevisiae*, secondary metabolites, free radicals, bacterial pathogen

## Abstract

**Research background:**

Antioxidants are important compounds present at low concentrations that inhibit oxidation processes. Due to the side effects of synthetic antioxidants, research interest has increased considerably towards finding natural sources of antioxidants that can replace the synthetic ones. The emergence and spread of antibiotic resistance require the development of new drugs or some potential sources of novel medicine. This work aims to extract the secondary metabolites of *Saccharomyces cerevisiae* using ethyl acetate as a solvent and to determine the antioxidant and antimicrobial activities of these extracted metabolites.

**Experimental approach:**

The antioxidant activity of the secondary metabolites of *S. cerevisiae* were determined using DPPH, ABTS and FRAP assays. Furthermore, the antimicrobial potential of the ethyl acetate extract of *S. cerevisiae* against *Cutibacterium acnes*, *Staphylococcus aureus* and *Staphylococcus epidermidis* was assessed.

**Results and conclusion:**

Five out of 13 of the extracted secondary metabolites were identified as antioxidants. The antioxidant activity of the *S. cerevisiae* extract exhibited relatively high IC_50_ of 455.26 and 294.51 μg/mL for DPPH and ABTS respectively, while the obtained FRAP value, expressed as ascorbic acid equivalents, was 44.40 μg/mL. Moreover, the extract had a significant antibacterial activity (p<0.05) against *Staphylococcus aureus* and *Staphylococcus epidermidis* at the concentrations of 100 and 200 mg/mL, respectively. However, no inhibitory effect was observed against *Cutibacterium acnes* as the extract was only effective against the bacterium at the concentrations of 300 and 400 mg/mL (inhibition zones ranging from 9.0±0.0 to 9.3±0.6) respectively (p<0.05). *Staphylococcus aureus* was highly sensitive to the extract, with a MIC value of 18.75 mg/mL.

**Novelty and scientific contribution:**

This report confirmed the efficacy of the secondary metabolites of *S. cerevisiae* as a natural source of antioxidants and antimicrobials and suggested the possibility of employing them in drugs for the treatment of infectious diseases caused by the tested microorganisms.

## INTRODUCTION

*Saccharomyces cerevisiae* is a eukaryotic microbe that belongs to the Saccharomycotina family. Yeasts, including *S. cerevisiae,* possess the ability to produce antimicrobial and antifungal compounds that inhibit the growth of pathogenic bacteria and fungi (*1*). It produces toxic proteins or glycoproteins to combat other strains of yeast or bacteria (*2*). According to Hassan (*3*), glutathione (GSH), sulfur-containing amino acids and Maillard reaction products are the components that contribute to the antioxidative properties of *S. cerevisiae*. GSH is the most abundant thiol in yeast cells that plays a considerable role in antiradical activity (*4*). Besides that, Meng *et al.* (*5* ) reported that GSH and ascorbic acid can act as radical scavengers in *S. cerevisiae*. Ascorbic acid is a small water-soluble molecule that works with GSH to form a redox couple (*6*). GSH is described as a cofactor of oxidative stress enzymes that can modulate enzyme activity to maintain redox balance (*4*).

In recent years, natural antioxidants are becoming more likely to serve as alternatives to synthetic antioxidants probably due to the associated side effects of the synthetic antioxidants, such as their carcinogenicity and toxicity. Thus, there has been much interest in finding safer and more effective natural antioxidant sources (*7*). Secondary metabolites of *S. cerevisiae* have attracted more attention as a potential source of natural antioxidants owing to their strong bioactive properties in the human body (*8*). The use of *S. cerevisiae* as a safe source of ingredients and additives in food processing has been widely accepted by the consumers (*9*). Perhaps these secondary metabolites of *S. cerevisiae* can act as natural antioxidants; therefore, it is crucial to evaluate their antioxidant activity for potential application in the food and pharmaceutical industries.

To date, there are only a few yeast strains that can be referred to as producers of secondary metabolites with antioxidant properties. *Cutibacterium acnes*, a Gram-positive anaerobic bacterium, is believed to be the main causative agent of acne. Furthermore, *Staphylococcus epidermidis* and *Staphylococcus aureus* have been reported as the causative agents of acne vulgaris and have been isolated in 53 and 41% aerobic cultures of pustular and nodulocystic skin lesions, respectively (*10*). Different acne treatments include lifestyle remedies, topical medication, oral medication and medical procedures. However, patients may suffer from side effects from these treatments; hence, people nowadays prefer natural products as treatment options due to their body tolerance. This research focuses on the extraction and identification of the secondary metabolites of *S. cerevisiae*. Different antioxidant assays, namely DPPH, ABTS and FRAP, were employed to determine the antioxidant activity of the extracted metabolites. Finally, the antibacterial activity of the secondary metabolites of *S. cerevisiae* was investigated against *Cutibacterium acnes*, *Staphylococcus aureus* and *Staphylococcus epidermidis*.

## MATERIALS AND METHODS

### Materials

Potato dextrose agar (PDA), ethyl acetate, methanol, ascorbic acid, potassium persulfate hydrochloric acid iron(III) chloride hexahydrate (FeCl_3_·6H_2_O), dimethylsulfoxide (DMSO), Mueller-Hinton agar (MHA), tryptic soy agar (TSA), sterile Mueller-Hinton broth (MHB), and sterile tryptic soy broth (TSB) were purchased from Merck (Darmstadt, Germany). Potato dextrose broth (PDB) was obtained from CONDA (Madrid, Spain). DPPH solution, Tris(2-pyridyl)-*s*-triazine (TPTZ) and iodonitrophenyltetrazolium violet were purchased from Sigma-Aldrich, Merck (St Louis, MO, USA). ABTS powder was obtained from Roche (Basel, Switzerland).

### Yeast cultivation

Potato dextrose agar (PDA) was prepared by dissolving 39 g dehydrated medium in 1 L distilled water. The medium was stirred, heated and sterilized by autoclaving at 121 °C for 15 min. The medium was then poured into agar plates and allowed to solidify. The yeast *Saccharomyces cerevisiae* was obtained from the laboratory unit of the Faculty of Industrial Sciences and Technology (FIST), Universiti Malaysia Pahang (UMP). The yeast was streaked on PDA with a sterile inoculating loop and incubated at 30 °C for 3 days. Potato dextrose broth (PDB) was prepared by dissolving 26.5 g dehydrated medium in 1 L distilled water. The medium was stirred, heated and autoclaved at 121 °C for 15 min. A single yeast colony was picked from the PDA and cultivated in the PDB, followed by incubation in an orbital shaking incubator (BD115; Binder, Tuttlingen, Germany) for 3 days at 25-30 °C with mild agitation at 130 rpm.

### Extraction of secondary metabolites from yeast

The extraction was carried out according to the method described by Swathi *et al.* (*11*). Ethyl acetate was added as a solvent to the yeast liquid culture in 1:1 ratio. The mixture was shaken for 10 min in a separatory funnel for complete extraction of the secondary metabolites. Then, it was allowed to settle for a few minutes. Two layers of liquid were formed; the upper layer containing the metabolites was collected into Falcon tubes, while the bottom layer containing the yeast cells and PDB was washed thoroughly to ensure complete extraction of the metabolites before being discarded. The separated upper layer was centrifuged at 2900×*g* for 5 min (centrifuge Rotofix 32; Hettich, Schwerin, Germany) to remove any suspended yeast cells and medium contaminants. The resulting supernatant was collected and evaporated to dryness at 35-40 ºC using an RV 10 digital rotary evaporator (N-1200A; Evela, Shanghai, PR China). A green-coloured extract was obtained after the drying and stored for further use.

### Analysis of secondary metabolites of yeast using GC-MS

The crude extract was diluted in gas chromatography (GC) grade ethyl acetate before GC-MS analysis. The analysis was conducted using a GC-MS 6890A system (Agilent, Santa Clara, CA, USA). A volume of 1 μL of the sample was introduced into the heated injector tube using a microliter volume syringe. The vapourised sample was carried through the SGE BPX5 GC column by helium gas at the rate of 1.0 mL/min. The components in the sample were separated and a gas chromatogram was obtained. Then, the effluent of the GC column was introduced directly into the mass spectrometer *via* a transfer line at 320 °C. The gas molecules were converted into ions at an ion source temperature of 230 °C using electron energy of 70 eV. The scan range was set at 45-500 Da. The ions were detected by a detector and the information was sent to the computer. The components were identified based on their retention indices and by comparison of their mass spectra with the available data in the existing GC-MS library (NIST/NIH/EPA mass spectral library[REMOVED HYPERLINK FIELD]) (*12*).

### Determination of antioxidant activity

#### DPPH assay

DPPH assay was conducted according to the suggested method by Hassan (*3*) with some modifications. DPPH solution (0.1 mM) was first prepared in absolute methanol and added into different tubes at 1 mL volumes. Then, 1.0 mL yeast extract was added into each tube at different concentrations (125-2000 μg/mL) and mixed well. A set of blanks was prepared by adding 1.0 mL methanol with 1.0 mL yeast extract at different concentrations. All the mixtures were incubated in the dark at room temperature for 30 min. Negative control was prepared by the same procedure without the yeast extract. Ascorbic acid solution was used as a positive control. The absorbance of the resulting mixtures was measured at 517 nm using a 10S UV-Vis spectrophotometer (Genesys, Daly City, CA, USA). The measurements were taken in triplicate and the mean values were calculated.

#### ABTS assay

The ABTS assay was carried out based on the method proposed by Hameed *et al.* (*13*) with some modifications. The stock solution was prepared by reacting the 7.00 mM ABTS solution with 2.45 mM potassium persulfate at the ratio of 1:1 (*V*/*V*), and kept in the dark at room temperature for 12-16 h before use (to generate ABTS˙^+^). The ABTS˙^+^ solution was diluted with absolute methanol and adjusted to the absorbance from 0.7 to 0.75 at 734 nm. A volume of 200 µL of the yeast extract at different concentrations (15.63-2000 µg/mL) and 1.8 mL ABTS˙^+^ solution were mixed well in the tubes and incubated in the dark at room temperature for 30 min. Negative control was prepared according to the same procedure without the yeast extract. Ascorbic acid solution was used as a positive control. The absorbance was measured at 734 nm using Infinite 200 PRO microplate reader (Tecan, Männedorf, Switzerland). The measurements were taken in triplicate and the mean values were calculated.

#### FRAP assay

The FRAP reagent was prepared by mixing 300 mM acetate buffer at pH=3.6, 10 mM TPTZ in 40 mM hydrochloric acid (Merck), and 20 mM FeCl_3_·6H_2_O at the ratio 10:1:1. The mixture was incubated for 15 min at 37 °C before use. Ascorbic acid was used in distilled water at different concentrations (200-1000 μg/mL) as the positive control to generate a standard curve. A volume of 150 μL of the yeast extract (in ethyl acetate) or standard was mixed thoroughly with 2.85 mL FRAP reagent in Falcon tubes. The formation of an intense blue colour complex suggests the reduction of Fe^3+^ to Fe^2+^ in TPTZ complex. The mixture was incubated in the dark for 30 min. The absorbance (*A*) of the solution was measured at 593 nm using the Infinite 200 PRO microplate reader (Tecan). The measurements were taken in triplicate and the mean values were calculated.

### Evaluation of antibacterial activity of yeast secondary metabolites

#### Kirby-Bauer disc diffusion susceptibility test

In this test, the yeast extract sample was used against *Cutibacterium acnes* (ATCC 6919), *Staphylococcus aureus* (ATCC 6538) and *Staphylococcus epidermidis* (ATCC 12228). The concentrations of the yeast secondary metabolites were 100, 200, 300 and 400 mg/mL. The extract was dissolved in DMSO. Gentamycin discs (Thermo Scientific™ Oxoid™) with 10 µg drug acted as the positive control, while the disc with DMSO solvent but without any extract acted as the negative control. A volume of 7 µL of the dissolved extract and solvent was impregnated into sterile discs and dried in the laminar flow hood (model AHC-4DI; ESCO, Selangor, Malaysia) to remove the solvent completely. The antimicrobial tests against *S. aureus* and *S. epidermidis* were carried out in MHA, while the disc diffusion test against *C. acnes* was carried out on TSA as *C. acnes* cannot grow on MHA. The three tested bacterial strains were cultured in broth and the concentration of the bacteria was adjusted to 0.5 McFarland standard. According to Clinical and Laboratory Standards Institute (CLSI), 0.5 McFarland standard is equivalent to an absorbance value range from 0.08 to 0.13 at 625 nm. The TSA plates with *C. acnes* were incubated in the anaerobic jar at 37 °C for 48-72 h, while the MHA plates were incubated in the presence of oxygen at 37 °C for 8-16 h. The formed inhibition zones were observed after every 6 h of incubation; the diameter of the zones was measured in mm. This test was performed in triplicate.

#### Determination of minimum inhibitory concentration

The minimum inhibitory concentration (MIC) of the extracts was determined in a 96-round bottom well microplate. The initial concentration of the secondary metabolites of *S. cerevisiae* used for the MIC determination was the lowest concentration that formed an inhibition zone in the disc diffusion method for the three bacterial strains. Two-fold serial dilution was carried out to make a total of eight different concentrations of the extract. Sterile MHB was added to each well containing *S. aureus* or *S. epidermidis*, while sterile TSB was added for *C. acnes*. The extract was prepared in 100% DMSO at the concentration of 300 mg/µL, then 100 µL of the prepared extract were transferred to the first column of the well, mixed, and 100 µL of the mixture were also transferred to the next well in each row. Gentamicin was used as the positive control at the concentration of 1 mg/µL, while the negative control was the broth and solvent without any extract. The suspension of the three tested bacterial strains was adjusted to the concentration equivalent to 0.5 McFarland standard using a UV-Vis spectrophotometer (Genesys). A volume of 100 µL bacterial suspension was added into each well, then the microplate was covered, sealed, and incubated at 37 °C. The microplates containing *S. aureus* and *S. epidermidis* were then incubated for 6 h and those containing *C. acnes* 12 h. After the incubation period, 50 µL of 0.4 mg/mL *p*-iodonitrophenyltetrazolium violet indicator (Sigma-Aldrich, Merck) was added into each well and further incubated for 30 min. The colour change of each well was observed. The colour of the indicator turns pink when there is active bacterial growth in any well, while no colour change indicates no active bacterial growth. The lowest concentration of the extract that inhibited bacterial growth was recorded as the MIC. This assay was performed in triplicate.

### Statistical analysis

Statistical analysis of the obtained data was carried out using one way-ANOVA. The mean value and standard deviation of the data were generated with the level of significance set at p<0.05.

## RESULTS AND DISCUSSION

### Extraction of S. cerevisiae secondary metabolites and GC-MS analysis

The selection of a solvent for extraction depends on the nature of the desired bioactive compounds. Ethyl acetate, which was used as an extraction solvent in this study, has a medium polarity with a polarity index of 4.4. It has low toxicity on the tested strains and mild effect on biological cells (*14*). When the liquid broth cultured with *S. cerevisiae* was mixed with 100% ethyl acetate in the separatory funnel, two distinct layers formed as ethyl acetate is immiscible with water. Generally, organic compounds do dissolve in organic solvents. Ethyl acetate can even extract compounds from the aqueous layer. The GC-MS results were obtained by comparing the spectra and matching them against the library database (NIST/NIH/EPA mass spectral library (*12*)). Table 1 shows the secondary metabolites recognized in *S. cerevisiae* extract.

**Table 1:** Secondary metabolites identified in the ethyl acetate extract of *S. cerevisiae*

**Compound**	**Molecular formula**	***t_R_*/min**	**Peak area/%**
Benzeneethanol,4 hydroxy-	C_8_H_10_O_2_	11.548	12.14
1-Methyl-3,3-diphenylurea	C_14_H_14_N_2_O	13.796	1.26
1H-Indole-3-ethanol	C_10_H_11_NO	15.505	10.68
Diethyldithiophosphinic acid	C_4_H_11_PS_2_	16.082	2.09
9-Hexadecenoic acid	C_16_H_30_O_2_	17.020	1.66
Diethyldithiophosphinic acid	C_4_H_11_PS_2_	17.057	1.90
2-Hydroxy-3,5,5-trimethyl-cyclohex-2-enone	C_9_H_14_O_2_	17.230	1.80
Hexadecanoic acid, butyl ester	C_20_H_40_O_2_	19.133	1.15
Octadecanoic acid, butyl ester	C_22_H_44_O_2_	20.852	0.69
Pyrrolo[1,2-a]pyrazine-1,4-dione,hexahydro-3-(phenylmethyl)-	C_14_H_16_N_2_O_2_	21.444	3.74
1,2-Benzenedicarboxylic acid, mono(2-ethylhexyl) ester	C_16_H_22_O_4_	22.152	58.33
2,3-Diphenyl-5,8-dimethoxy-6-acetamidoquinoxaline	C_24_H_21_N_3_O_3_	23.117	1.21
l-Proline, N-allyloxycarbonyl-, undec-10-enyl ester	C_20_H_33_NO_4_	24.579	3.35

Fig. S1 shows the chromatogram of the extracted secondary metabolites from *S. cerevisiae*. Based on the results, the longest retention time of 24.58 min was recorded for l-proline, *N*-allyloxycarbonyl-, undec-10-enyl ester. The retention time of any compound depends on the different strengths of its interaction with the stationary phase. Thus, this compound had the strongest interaction with the stationary phase and required more time to migrate through the column. The 1,2-benzenedicarboxylic acid mono(2-ethylhexyl) ester had the highest concentration in the extract of *S. cerevisiae* with 58.33% of the total peak area. The GC-MS analysis of *Saccharomyces cerevisiae* has been reported to identify the presence of different compounds, such as thieno[2,3-c]furan-3-carbonitrile, 2-amino-4,6-dihydro-4,4,6,6-, oxime-, methoxyphenylacetic acid, erythritol, 3-[3-bromophenyl]-7-chloro-3,4-dihydro-10-hydroxy-1,9 (2H,10H), 2-methyl-9-β-d-ribofuranosylhypoxanthine, dodecane,1-chloro-, 2,7-diphenyl-1,6-dioxopyridazino [4,5:2',3'] pyrrolo[4',5'-d] pyridazine, and 2-bromotetradecanoic acid (*15*).

### Antioxidant activity

#### DPPH radical scavenging activity

**Fig. 1:**
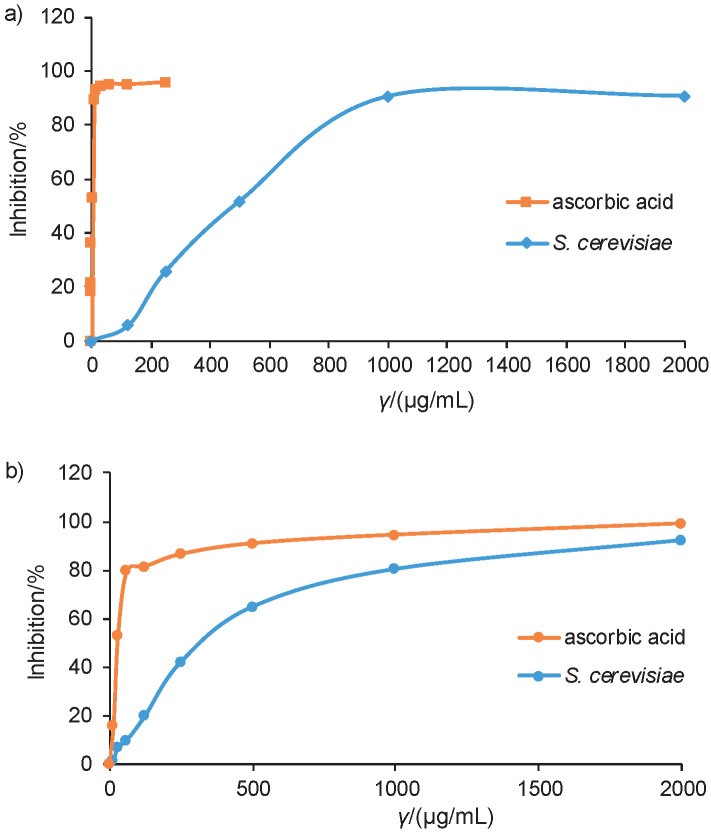
Antioxidant assays: a) DPPH radical scavenging activity of standard ascorbic acid and Saccharomyces cerevisiae extract, and b) ABTS radical scavenging activity of ascorbic acid and S. cerevisiae extract

The DPPH radical scavenging activity of the *S. cerevisiae* extract was evaluated based on its ability to reduce DPPH free radicals, which decolourises purple DPPH solution (*16*). Fig.1a shows the radical scavenging ability of the *S. cerevisiae* extract compared to ascorbic acid. The highest DPPH radical scavenging activity of ascorbic acid and the yeast extract was 95.91 and 90.96% at the concentrations of 250 and 2000 μg/mL, respectively. Ascorbic acid exhibited a significantly higher DPPH radical scavenging activity than the *S. cerevisiae* extract. Ascorbic acid is one of the active reducing agents and scavengers of free radicals in biological systems, acting as a scavenger of free oxidizing radicals and harmful oxygen species (*17*). Hassan (*3*) documented that 25 mg/mL baker’s yeast autolysate showed (69.06±1.22) % DPPH radical scavenging activity due to the reduction of the molarity of the DPPH solution from 0.2 to 0.1 mM. Moreover, the extraction method might affect the antioxidant capacity of an extract (*18*). The study by Sugiyama *et al*. (*19*) evaluated the antioxidant activity of indole derivatives from a marine sponge-derived yeast. All compounds showed weak activities in the DPPH assay. The extract of *S. cerevisiae* has been reported to exhibit the highest DPPH radical scavenging activity due to its high tryptophol content from alcoholic fermentation (*20*). Ethanolic extracts of *Hibiscus sabdariffa* and *Croton caudatus* leaves have been evaluated for free radical scavenging activity in the model system of *S. cerevisiae*. *H. sabdariffa* and *C. caudatus* at a concentration of 500 μg/mL demonstrated an immense free radical scavenging capacity of DPPH with an IC_50_ value of 184.88 and 305.39 μg/mL, respectively (*21*). Furthermore, it has been demonstrated that soybean with *Tricholoma matsutake* and *S. cerevisiae* showed considerable DPPH radical scavenging activity (*22*).

#### ABTS radical scavenging activity

The ABTS radical scavenging activity of the *S. cerevisiae* extract was assessed based on its ability to reduce ABTS radical cation. The ABTS˙^+^ was produced from the oxidation of ABTS with potassium persulfate, which involves the loss of an electron from the nitrogen atom of ABTS (*23*). The ABTS˙^+^ solution is bluish-green in methanol. Its absorbance was determined at 734 nm to minimize the interference from other absorbing components and sample turbidity. Fig. 1b shows the relative ability of the *S. cerevisiae* extract to scavenge ABTS˙^+^ compared to that of ascorbic acid. The standard ascorbic acid and yeast extract at the concentration of 2000 μg/mL recorded the highest inhibition values of 99.17 and 92.20% respectively. This result indicated that both ascorbic acid and yeast extract effectively inhibited the formation of ABTS˙^ +^. It has been reported that endophytic yeasts isolated from the lower stem and roots of *Phragmites australis* Cav showed 88% radical scavenging activity in the ABTS assay (*24*). Furthermore, *Aspergillus awamori* DT11 exhibited ABTS˙^+^ scavenging activity of 34.07% compared to ascorbic acid (44.5%), which was used as a positive control (*25*).

#### Results of FRAP assay

The FRAP of *S. cerevisiae* extract was evaluated based on the ability to reduce iron(III) to iron(II)) *via* electron donation to the sample. This reduction was monitored by measuring the change in the absorbance at 593 nm. The assay was conducted at low pH values (pH=3.6) to maintain iron solubility and electron transfer (*26*). Table 2 shows the FRAP values of each yeast extract at different concentrations. In general, the absorbance readings increased when the concentration of ascorbic acid and yeast extract increased from 200 to 1000 μg/mL. This was due to the reduction of the Fe^3+^ in TPTZ complex to the intensely blue-coloured Fe^2+^ (*26*). The standard solution of ascorbic acid and yeast extract recorded the highest absorbance of 3.6291 and 0.1611 respectively, at the concentration of 1000 μg/mL. The yeast extract exhibited the highest FRAP value expressed as ascorbic acid equivalents of 44.40 μg/mL, indicating that *S. cerevisiae* can act as a potent antioxidant to reduce Fe^3+^ ion. Trolox, a water-soluble vitamin E analogue, was used as a reference standard to determine the antioxidant capacity of yeast microcarriers. The Trolox equivalent values for yeast cell wall particles and native yeast were reported to be 0.20 and 7.48 μM, respectively (*27*). Chen *et al.* (*28*) reported that *Lactobacillus rhamnosus* and *Saccharomyces cerevisiae* significantly improved FRAP in comparison to their non-fermented counterparts, and both activities were attributed to the released phenolics during the solid-state fermentation.

**Table 2:** FRAP values obtained for different yeast extract concentrations

*γ*(yeast extract)/(μg/mL)	*A*	FRAP(as AAE)/(μg/mL)
0	0.0000	0
200	0.0402±0.0007	36.73
400	0.0742±0.0039	39.10
600	0.1130±0.0075	43.11
800	0.1364±0.0006	43.98
1000	0.1611±0.0041	44.40

AAE=ascorbic acid equivalents

Benzeneethanol, 4-hydroxy, known as tyrosol, showed antioxidant activity *in vitro* and was able to inhibit or slow down the oxidation reactions brought by dioxygen or peroxides in animal tissues (*29*). However, tyrosol with its derivatives appeared to be less active than hydroxytyrosol and its analogues due to the influence of catechol structure (*29*). Antenucci *et al.* (*30*) reported that oligotyrosol exhibited higher antioxidant activity than tyrosol using DPPH, FRAP and hydroxyl radical scavenging assays. 1H-indole-3-ethanol is indolyl alcohol substituted with a 1H-indol-3-yl group. 1H-indole-3-ethanol plays a role as a metabolite of both plants and *S. cerevisiae*. Hexadecanoic acid is a fatty acid ester of plant and animal origins that acts as an insect repellent. Extracted from wild-growing mushrooms, it exhibited antioxidant activity at the concentration of 0.10 mg/mL (*31*). It is one of the twenty bioactive compounds isolated from *Thesium humile* Vahl with reported antioxidant activity (*32*). Gondwal *et al.* (*33*) reported that the water extract from the seeds and fruit pulp of *Skimmia anquetilia* can be used as a natural antioxidant due to its hexadecanoic acid content. Octadecanoic acid is a fatty acid ester and an algal metabolite that acts as a defoaming agent in processing beet sugar and yeast. Hexane extract of *Sinapis alba* exhibited slightly higher antioxidant activity than the standard ascorbic acid in phosphomolybdenum assay due to the existence of octadecanoic acid and other phytocomponents (*34*). Pyrrolo[1,2-*a*]pyrazine-1,4-dione, hexahydro isolated from a marine bacterium *Bacillus tequilensis* MSI45 exhibited high antioxidant activity (*35*). The antioxidant activity of different pyrrolizidines, including pyrrolo[1,2-a]pyrazine-1,4-dione, and hexahydro-3-(phenylmethyl) in the ethyl acetate extract of *Streptomyces omiyaensis* has been reported by Tangjitjaroenkun (*36*). Also, *Streptomyces* strain MUSC 149(T) showed a strong antioxidant activity due to its pyrrolo[1,2-a]pyrazine-1,4-dione, hexahydro- content (*37*).

### Antimicrobial activity

Disc diffusion method is a popular antimicrobial test in microbiology laboratories due to its simplicity and ability to test multiple antimicrobial agents (*38*). *S. epidermidis* and *S. aureus* can grow in a range of pH=4.0-9.8, with an optimum pH=6-7 (*39*). *C. acnes* showed optimal growth in the range from pH=6.0 to 7.0; however, it can grow in the range pH=5.0 to 8.0 (*39*). The pH value of the dissolved extract ranged from 6 to 7, which is considered suitable for bacterial growth. Four concentrations of the dissolved extract (100, 200, 300 and 400 mg/mL) were used in this assay. The dry mass of the extract loaded onto sterile discs was 0.7, 1.4, 2.1 and 2.9 mg. The observed antibacterial activity of the *S. cerevisiae* extract is shown in Table 3 . The results revealed that *S. cerevisiae* extract effectively suppressed the growth of the tested bacteria at different potency levels.

**Table 3:** Antimicrobial screening test of *Saccharomyces cerevisiae* extract against some bacterial strains

**Pathogenic bacteria**	***d*(inhibition)/mm**				
*γ*(extract)/(mg/mL)				*m*(gentamycin)=10 µg
100	200	300	400	
*S. aureus*	9.5±2.1	10.0±1.4	11.0±1.4	11.5±2.1	18.7±0.6
*S. epidermidis*	8.5±0.7	8.5±0.7	10.0±0.0	10.5±0.7	22.3±0.6
*C. acnes*	0.0	0.0	9.0±0.0	9.3±0.6	12.0±0.0

Values are expressed as mean±SEM. Analysis was performed with one-way ANOVA, where p<0.05

Among the tested bacteria, both *S. aureus* and *S. epidermidis* were susceptible to 400 mg/mL *S. cerevisiae* extract. As shown in Table 3, the most susceptible bacterium to 400 mg/mL extract was *S. aureus* (11.5 mm), followed by *S. epidermidis* and *C. acnes* with mean inhibition zones of 10.5 mm and 9.3, respectively. At the concentrations of 100 and 200 mg/mL *S. cerevisiae* extract, *C. acnes* had no inhibition zone, whereas the other bacteria show mean zones of inhibition in the range from 8.5 to 10.0 mm. The inhibition zones of the extract at 100 and 200 mg/mL were significantly different from those of the positive control (gentamycin 10 µg; p<0.05) for all the tested bacteria except for *C. acnes*. Moreover, the inhibition zones of the extract at 100 and 200 mg/mL were significantly different from that at 300 and 400 mg/mL for *S. aureus* and *S. epidermidis* (p<0.05). Based on the mean value of the zones of inhibition, the antibacterial activity of the *S. cerevisiae* extract was concentration-dependent. Al-Jassani *et al*. (*15*) found the inhibition zones of *S. cerevisiae* extract at the volume of 90 μL to be 5.33 and 5.10 mm for *S. epidermidis* and *S. aureus,* respectively. *S. cerevisiae* is employed as a human probiotic and its effects on the host’s health include antimicrobial, nutritional, trophic, immunomodulatory, anti-inflammatory, quorum sensing, inactivation of bacterial toxins, maintenance of epithelial barrier integrity and cell restitution (*40*). Moderate antimicrobial activity of *S. cerevisiae* against bacteria and fungi has been documented; furthermore, better antimicrobial activity of cell lysate than of whole-cell and culture supernatant has been shown. Again, the isolate showed better antibacterial activity against Gram-negative than Gram-positive bacteria (*41*). Additionally, *S. cerevisiae* appeared to have bacterial activity against *Pseudomonas* sp., *Salmonella* sp., *E. coli*, *Vibrio cholera* and *Staphylococcus aureus* (*42*). Chen *et al*. (*43*) evaluated the antimicrobial activity of *S. cerevisiae* through the inhibition of the growth of pathogenic *E. coli* O8 (MIC=0.025 g/mL), as well as its influence on the characteristics of its cell surface. *C. intermedia*, *C. kefyr,* and *C. lusitaniae* exhibited high antimicrobial activity against *E coli*, while *C. tropicalis*, *C. lusitaniae* and *S. cerevisiae* showed moderate antimicrobial activity against *E. coli*. However, all the tested yeasts demonstrated a very low activity against *P. aeruginosa* (*44*). Benzeneethanol,4-hydroxy- is one of the identified metabolites of *S. cerevisiae* that possess antibacterial activity against human pathogenic bacteria. 1-Methyl-3,3-diphenylurea and 1H-indole-3- have also been reported to possess antimicrobial, cardioprotective and anticarcinogenic properties. Diethyldithiophosphinic acid, known as O,O-diethyl dithiophosphate, has been shown to inhibit the growth of *E. coli, S. aureus* and *Aspergillus fumigatus* (*45*). The 9-hexadecenoic acid, present in *J. curcas* leaf extracts, has been shown to possess antimicrobial properties (*46*). Many fatty acids, such as hexadecanoic and octadecanoic acid, have been documented to show antibacterial and antifungal activities (*47* ,*48*). Pyrrolo[1,2-*a*]pyrazine-1,4-dione,hexahydro isolated from a marine bacterium *Bacillus tequilensis* exhibited a potent inhibitory effect against multidrug-resistant *S. aureus* (MIC=15±0.172) mg/L and (MBC=20±0.072) mg/L (*35*). Furthermore, pyrrolo[1,2-*a*]pyrazine-1,4-dione,hexahydro-3-(phenylmethyl)- (PPDHP) extracted from *Streptomyces* sp. has reported antifungal activity (*49*).

#### Minimum inhibitory concentration of *S. cerevisiae* extract

**Fig. 2:**
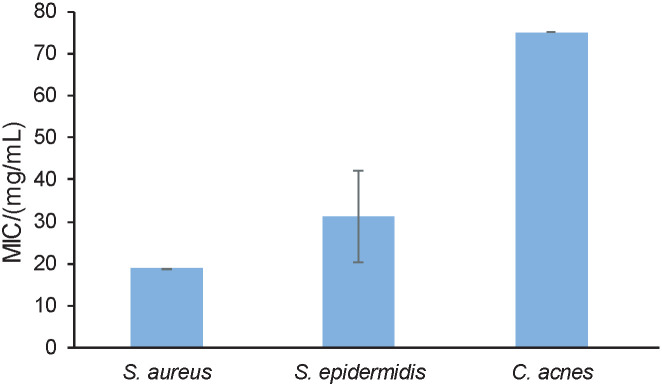
Minimum inhibition concentration (MIC) of *Saccharomyces cerevisiae* extract against the tested microbes

The MIC of *S. cerevisiae* extract was determined using a colorimetric method in which the clear-cut end-points were determined *via* a colour change. Visual detection of bacterial growth *via* turbidity or pellet formation in the wells may be difficult and could lead to inaccurate results (*50*). To detect the presence of bacterial growth, *p*-iodonitrophenyltetrazolium violet was used as an indicator. When there is active bacterial growth, colour changes from light yellow to pink or violet (Fig. S2). The MIC of the extracted metabolites was 18.75, 31.25 and 75 mg/mL against *S. aureus*, *S. epidermidis* and *C. acnes,* respectively. This showed the potential of developing these metabolites into compounds with promising bioactivities against pathogenic microorganisms (Fig. 2). The obtained MIC of the extract against *S. aureus* showed consistency at 18.75 mg/mL in the triplicate tests. The mean MIC for *S. aureus* was the lowest among the three bacterial isolates, which indicates that the secondary metabolites of *S. cerevisiae* extract showed better antibacterial activity against *S. aureus* than the other two bacteria. *C. acnes* showed the highest level of resistance against both gentamycin and the tested secondary metabolites of *S. cerevisiae*.

## CONCLUSIONS

This study provides new scientific information about *Saccharomyces cerevisiae* based on the results of the analysis of its secondary metabolites, antioxidant and antibacterial potential. The secondary metabolites produced by *S. cerevisiae* can act as natural antioxidants and can be used in industrial and pharmaceutical applications. Additionally, the secondary metabolites extracted from *S. cerevisiae* had good antimicrobial activity against the tested pathogens. The antibacterial activity of *S. cerevisiae* may be attributed to the various phytochemical constituents present in the extract. The individual compounds responsible for this property can be used in cosmetic industry.
